# Photoprotection as a Trait for Rice Yield Improvement: Status and Prospects

**DOI:** 10.1186/s12284-015-0065-2

**Published:** 2015-09-30

**Authors:** Erik H. Murchie, Asgar Ali, Tiara Herman

**Affiliations:** Division of Plant and Crop Science, School of Biosciences, University of Nottingham, Sutton Bonington Campus, Leicestershire, LE12 5RD UK; School of Biosciences, University of Nottingham Malaysia Campus, Semenyih, 43500 Selangor Darul Ehsan Malaysia

**Keywords:** Rice, Photoprotection, Photosynthesis, Radiation, Chloroplast, Oxidative Stress, Photoinhibition

## Abstract

Solar radiation is essential for photosynthesis and global crop productivity but it is also variable in space and time, frequently being limiting or in excess of plant requirements depending on season, environment and microclimate. Photoprotective mechanisms at the chloroplast level help to avoid oxidative stress and photoinhibition, which is a light-induced reduction in photosynthetic quantum efficiency often caused by damage to photosystem II. There is convincing evidence that photoinhibition has a large impact on biomass production in crops and this may be especially high in rice, which is typically exposed to high tropical light levels. Thus far there has been little attention to photoinhibition as a target for improvement of crop yield. However, we now have sufficient evidence to examine avenues for alleviation of this particular stress and the physiological and genetic basis for improvement in rice and other crops. Here we examine this evidence and identify new areas for attention. In particular we discuss how photoprotective mechanisms must be optimised at both the molecular and the canopy level in order to coordinate with efficient photosynthetic regulation and realise an increased biomass and yield in rice.

## Introduction

As the only significant source of energy, incident solar radiation (sunlight) at the surface of the earth governs climate and the meteorological cycles and is essential for life. It has a major effect on the diurnal and seasonal temperature variations as well as the productivity of both terrestrial and aquatic plants by providing energy for the assimilation of carbon by plant canopies (Zhu et al. 2008b, Kambezidis et al. 2012). One of the most critical components affecting crop yield is the amount of solar radiation available to the plant, and the ability and efficiency with which biomass is produced (Russell et al. 1989; Murchie et al. 2009; Zhu et al. 2010). Indeed one of the cornerstones of crop productivity is the close relationship between intercepted radiation and biomass, which defines radiation use efficiency and maximum productivity (Monteith and Unsworth 1990; Murchie and Reynolds 2012). It is important for our understanding of crop productivity to incorporate spatial and temporal variation in light intensity. However, the reduction of quantum yield that results from the inactivation of photosynthesis during high light episodes (termed photoinhibition) is rarely considered in this context, despite evidence that it can significantly affect yield and fitness in crops and model plant species (Zhu et al. 2004; Frenkel et al. 2007; Raven 2011). This may be particularly relevant for rice growing regions in South and Southeast Asia, which are positioned close to the equator where radiation can reach very high levels between periods of cloud cover. Here, the average annual solar irradiation can exceed 1800 kWh/m^2^, typical of the dry seasons in the tropics (data available at SolarGis.info). As an example, regions such as Central Luzon in the Philippines and Central and East Java in Indonesia can receive accumulative annual solar irradiation of more than 2200 kWh m^−2^. In regions that are environmentally vulnerable, higher light intensities could result in damage to crops and a significant reduction in yield. Photoinhibition is usually considered as a short-term response to periods of high light although is affected by long-term responses to environmental conditions (acclimation). In order to place photoinhibition in context with agricultural productivity which occurs over longer time scales it would be beneficial to link it with long term weather data. In this short review we summarise the current evidence that high irradiance levels result in significant yield reductions in rice crops at the metabolic and canopy levels.

### Review: Photosynthesis Under High Light: the Good and the Bad

The physiological basis for high light ‘stress’ in plants originates within the process of energy absorption and transduction and the fact that there is a limited capacity for plants to utilise absorbed light energy. Photosynthesis is a complex physio-chemical process that converts light energy into chemical energy via the fixing of CO_2_ into sugars. Light is first absorbed by chlorophyll, located in the light-harvesting complexes of both photosystems I and II (PSI, PSII) within the thylakoid membrane. This creates resonance energy, which is then transferred through neighbouring chlorophyll molecules to photosystems. An excited chlorophyll *a* molecule can return to ground state after light absorption through several pathways – photochemistry (photosynthesis), thermal dissipation, chlorophyll fluorescence or formation of a reactive oxygen species (ROS). The latter two are much less likely than the former (Murchie and Lawson 2013). Electrons are transferred from PSII to PSI via the redox reactions within the electron transport chain, coupled with the transfer of H^+^ across the thylakoid membrane. The resulting NADPH and ATP are utilized in the Calvin-Benson cycle to reduce CO_2_ to organic compounds. However, the capacity of photosynthesis to utilise sunlight is limited, and more so in C3 plants such as rice. Moreover this is a highly erratic resource, fluctuating according to climate and season cycles and canopy position and it is clear that photosynthesis does not maintain a high efficiency during such transients leading to the risk of biomass loss and oxidative stress (Pearcy 1990; Murchie et al. 2009; Niinemets and Anten 2009).

#### Photoinhibition: Classic and Contemporary Views

Photoinhibition is a state describing the light-dependent decline in quantum yield and sometimes the photosynthetic capacity of photosynthetic organisms. It is common even in optimal conditions, especially under high light (Murchie et al. 1999) but is exacerbated by other physiological stresses such as drought (Raven 2011). It is usually considered to represent damage to the D1 protein within the PSII complex resulting in inactive reaction centres. A new D1 protein has to be synthesized and incorporated into the PSII core complex for PSII to regain functionality (Zhang et al. 2000; Aro et al. 2005). The balance between rate of damage and replacement of the D1 protein in the PSII reaction centre is important, with the rate of repair recently being highlighted (Murata et al. 2007). Repair of PSII is dependent on time, metabolic state and environmental conditions and is a limiting factor for the re-establishment of high quantum yield and photosynthesis (Aro et al. 2005; Takahashi and Badger 2011).

**Fig. 1:**
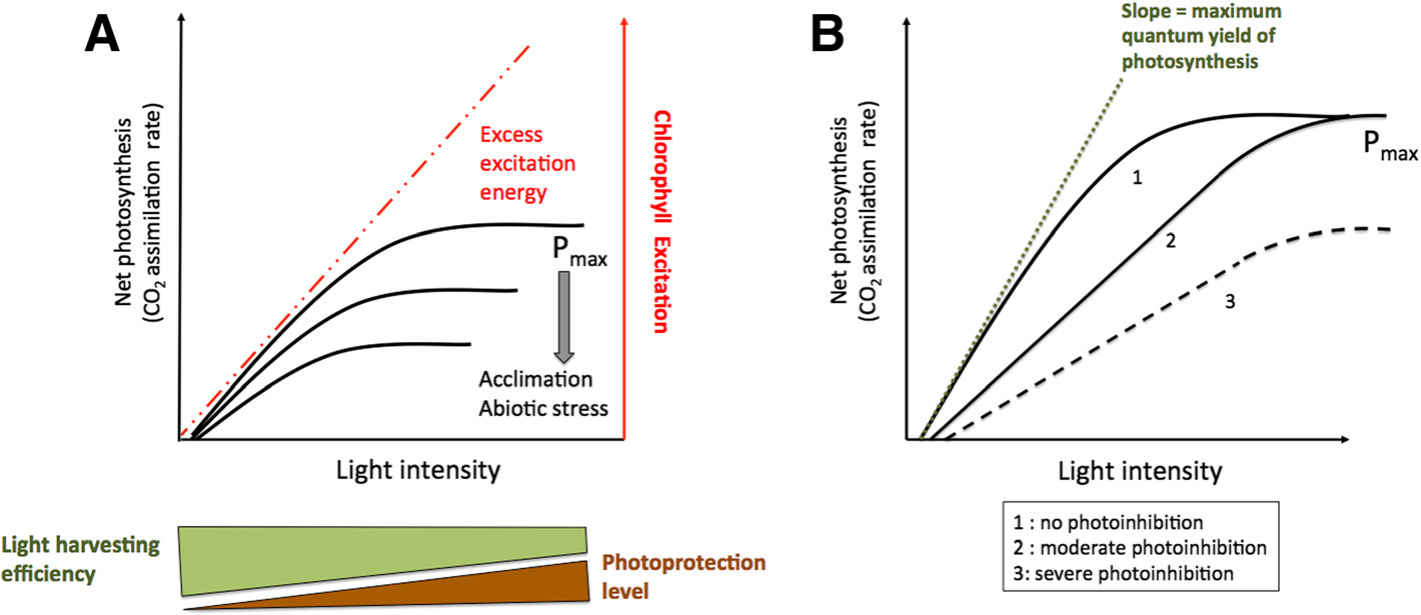
The impact of photoinhibition on leaf photosynthetic efficiency. **a**: schematic depiction of how excess excitation energy is formed by the saturation of CO_2_ assimilation and the continued absorption of irradiance. This results in a lowering of light harvesting efficiency under low light as photoprotective processes such as NPQ begin to form and reach a maximum under high light. The proportion of excess excitation energy rises as CO_2_ assimilation capacity falls. **b**: schematic depiction of the lowering of quantum yield and maximum photosynthetic capacity according to the severity of photoinhibition (adapted from Murchie and Niyogi 2011)

It may be difficult to separate the photosynthetic impact of photoinhibition with other metabolic perturbations caused by abiotic and biotic stresses. However it is clear that photoinhibition alone lowers ΦCO_2_, (quantum yield of CO_2_ assimilation) resulting in a sustained decline in photosynthesis (Fig. 1). Light experienced by leaves within a canopy fluctuates over space and time: canopy photosynthesis is therefore the result of large populations of chloroplasts at different states of light saturation. Thus reduction in ΦCO_2_ is visible during (temporary) low light episodes and can substantially limit photosynthesis (Ogren 1993; Zhu et al. 2004; Burgess et al. 2015). However, severe (chronic) photoinhibition can occur causing a decrease in both ΦCO_2_ and P_max_ (light-saturated photosynthesis) under very high light. While it has been suggested that under certain circumstances it may occur subsequent to photosynthetic down regulation or damage possibly associated with high carbohydrate levels (Adams et al. 2013), this is not consistent with observations of rice in the field (Murchie et al. 2002).

**Fig. 2:**
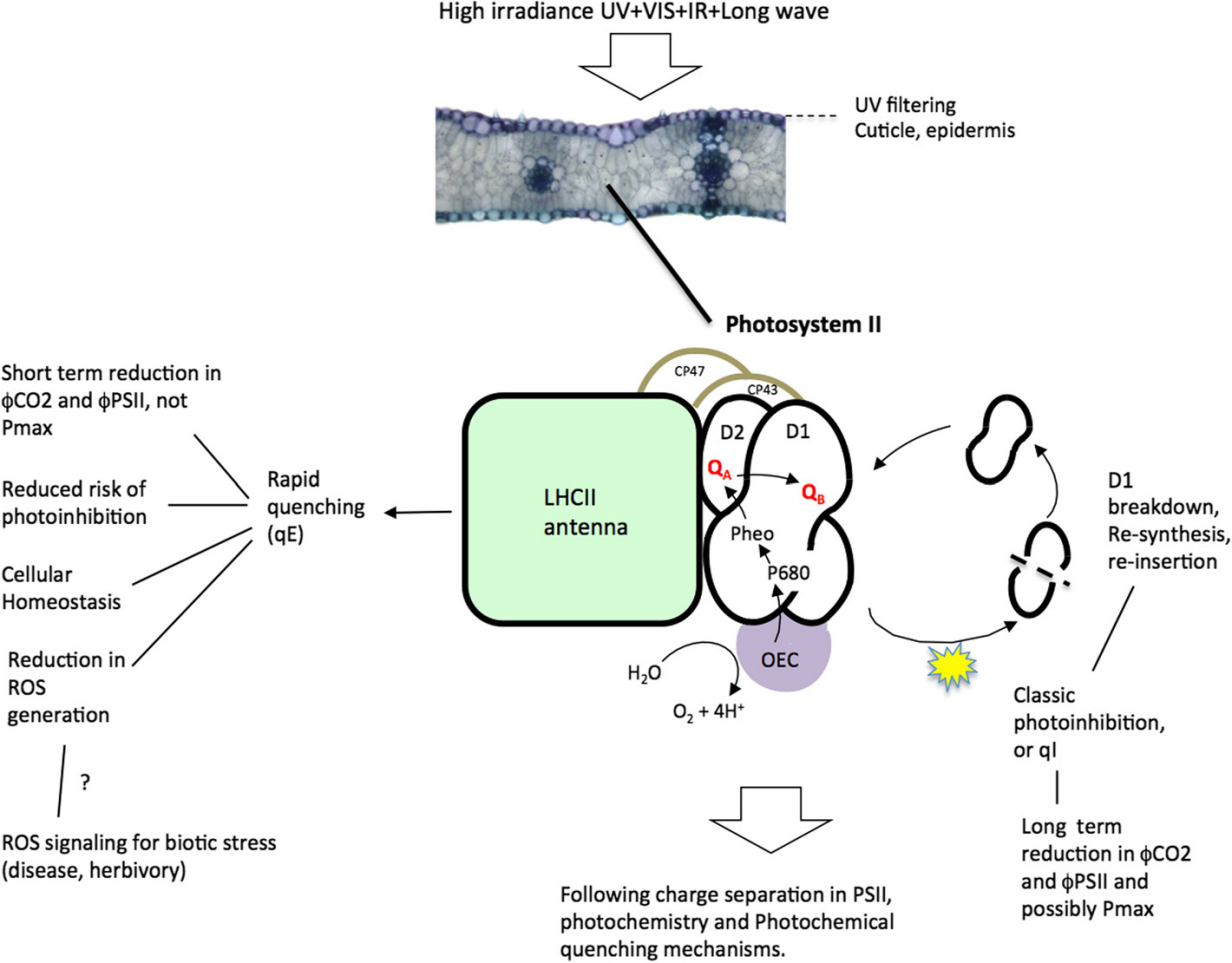
Summary of the major processes involved in protective NPQ and photoinhibition described in this mini-review. The objective of this figure is to help explain why the tradeoffs between photoprotection, photoinhibition and photosynthesis occur. D1, D2, CP47 and CP43 refer to PSII proteins. LHCII is the light harvesting complex associated with PSII and OEC refers to the oxygen evolving complex of PSII

Photoinhibition has fascinated plant scientists for over half a century and there have been many studies that conclude that there is an impact on plant productivity (Raven 1989; Ogren 1993; Werner et al. 2001). Despite this a complete empirical and mechanistic understanding of its impact on crop yield is still lacking (Long et al. 1994; Murchie et al. 2009). One of the reasons for this may be the established principle that crop biomass positively correlates with intercepted light leading to the perception that low light is a bigger influence on yield. However this view is limited because it ignores the slope of this relationship, the canopy radiation use efficiency. This is itself limited by a number of factors including photoinhibition (see below and discussed in depth in Murchie et al. 2009). Modelling the effects of a delayed recovery of ΦCO_2_ has consistently predicted a decline in canopy productivity as high as 30 % (Ogren 1993; Zhu et al. 2004). This lack of clarity could be due to the difficulty in routine measurement of photoinhibition and canopy photosynthesis over long periods and the great dependency on environmental and metabolic states, which can affect both the initiation of photoinhibition and the rate of recovery. Advances in technology and modelling, like remote fluorescence monitoring may overcome these limitations by the continuous automated logging of fluorescence at many points in the plant canopy (Porcar-Castell 2011; Ruban and Murchie 2012).

#### Photoprotection

In order to reduce photoinhibition, plants have evolved a cascade of adaptive mechanisms at both leaf and cellular levels (Fig. 2) (Demmig-Adams and Adams 2006; Murchie and Niyogi 2011). Photoprotection can occur via the avoidance of high light or the metabolic processing of absorbed light energy to avoid oxidative stress. Avoidance can involve the repositioning of leaves to minimise light absorption and altering the angle of incidence to enhance reflection (Murchie et al. 2009) or the movement of chloroplasts along cell walls (Wada et al. 2003). In this way, leaf angle may be a major factor in the onset of photoinhibition (Murchie et al. 1999). Accumulation of UV- absorbing compounds, for example in the leaf cuticle, may also contribute to photoprotection (Hakala-Yatkin et al. 2010; Takahashi et al. 2010).

There are many photoprotective mechanisms located within the thylakoid membrane and these can be broadly divided into photochemical (involve electron sinks) or non-photochemical (occur prior to photolysis in PSII). Non-photochemical quenching (NPQ) refers to the measurement of thermal dissipation of excess excitation energy in PSII (Horton and Ruban 2005; Belgio et al. 2014). The dominant form of NPQ is qE, a rapidly reversible type of NPQ induced by the pH gradient (ΔpH) across the thylakoid membrane in high light. Acidification of the thylakoid lumen leads to a change in protonation of the PSII proteins resulting in an increased probability of energy dissipation (Ruban et al. 2012). Protonation activates the xanthophyll cycle (XC) which consists of the de-epoxidation of the xanthophyll carotenoid, violaxanthin, to the intermediate antheraxanthin and finally to zeaxanthin. The XC has a critical role in determining the kinetics of rise and decay of NPQ in plant tissue with the reconversion to zeaxanthin taking several minutes depending on environmental conditions (Demmig-Adams and Adams 2006; Johnson et al. 2008). Zeaxanthin has also been shown to be a highly effective antioxidant (see below). Another critical component of NPQ formation, capacity and kinetics is the PsbS protein, originally thought to be required for qE formation but now considered to be mainly involved in the enhancement of the capacity and rate of formation of qE by inducing structural changes in the thylakoid (Johnson and Ruban 2010). There is little doubt that qE acts in a protective manner. In *Arabidopsis thaliana*, the impairment of qE quenching via the mutation of genes encoding the PsbS (*npq4*) proteins has been shown to accelerate photoinhibition under strong light (Li et al. 2002; Niyogi et al. 2005).

qE relaxes to zero within minutes of returning to darkness. A long-lived form of NPQ, labelled qI also exists and this can have multiple origins depending on the plant condition and environment (Horton et al. 2008). qI is also referred to as photoinhibition because it is measured the same way, i.e. as a sustained reduction in ΦPSII and ΦCO_2_ (Murchie and Lawson 2013). qI can also result from processes not associated with damage to PSII such as zeaxanthin retention (Demmig-Adams and Adams 2006). Since both qI and qE result in a decline in quantum yield they are sometimes referred to as chronic and dynamic photoinhibition respectively.

Any efficient sink for electrons may reduce the risk of photooxidative stress (see below) and photoinhibition. In fact, studies show a broad (negative) relationship that exists between the capacity to utilise light energy within photochemical sinks (carbon fixation and growth) and NPQ capacity (Demmig-Adams and Adams 2006). Fast growing species with a high capacity to generate biomass may have fewer requirements for protective quenching (see discussion below about photoinhibition and yield in rice). However the important point to make here is that besides photosynthesis itself, there are many mechanisms of photochemical quenching of excitation energy have been proposed including photorespiration, cyclic electron transport and the Mehler reaction (water-water cycle) (Murchie and Niyogi 2011).

Reactive oxygen species are readily generated in photosynthetic processes and this is central to the maintenance of photosynthetic efficiency and cellular signalling processes (Foyer and Noctor 2005; Murchie and Niyogi 2011). ROS are involved in the classical D1 turnover mechanism of photoinhibition described above and have been invoked in other mechanisms of PSII inactivation in different systems (Aro et al. 2005; Keren and Krieger-Liszkay 2011). Recent work places a greater importance on the function of ROS on the rate of repair of PSII and also a more specific role of NPQ in this regard (Takahashi and Badger 2011). Regulation of ROS could prove important in the control of photoinhibition in the field. Important antioxidants include carotenoids, α-tocopherol, glutathione and ascorbate. Johnson et al. (2007) showed that zeaxanthin, in addition to a key role in qE formation, was able to reduce lipid peroxidation in plants and improve whole plant tolerance to high light and temperature stress. A similar observation also made in rice (Du et al. 2010). α-tocopherol has recently been shown to be an important component of photoprotection in rice (Chen et al. 2014). Transgenic overexpressors of antioxidants such as glutathione reductase and superoxide dismutase (Foyer 1995), SOD (Kwon et al. 2002) and ascorbate peroxidase (Wang et al. 1999) have demonstrated the important role of radical scavenging. Generally there is a need to extend research into the role of such antioxidants in rice productivity (Du et al. 2010; Chai et al. 2011; Zulfugarov et al. 2014).

#### Photoinhibition in Rice

Rice cultivars exhibit varying degrees of susceptibility to photoinhibition and thermal dissipation that are likely to be a result of evolution across different geological regions (Jiao et al. 2003; Kasajima et al. 2011). In many cases tropical japonica cultivars are found to be more prone to photoinhibition (Gesch and Heilman 1999; Murchie et al. 1999; Murchie et al. 2002; Jiao et al. 2003; Zhu et al. 2008b) and there is evidence that these differences are associated with the ability to regulate active oxygen and membrane stability (Jiao et al. 1992; Jiao and Ji 2001; Zhu et al. 2008a). However there may be a wider range of metabolic and physiological factors involved in the field that compound the effect of high light (Black et al. 1995; Chen et al. 2003; Wang et al. 2005) . Clearly there is scope for improvement if the correct genetic resources can be applied to breeding. One example of this is the genetic analysis of NPQ across a rice core collection (Kasajima et al. 2011). Here, higher NPQ was associated with Japonica cultivars in comparison to Indica. Remarkably the locus was associated with a PsbS homologue suggesting this gene may be a candidate for the higher photoprotective capacity in this ecotype, consistent with other overexpression analyses in rice (Hubbart et al. 2012). It is unclear why this contrasts with a previous quantitative trait loci study in *A. thaliana* that failed to associate NPQ with presence or PsbS (Jung and Niyogi 2009). It now seems likely that that PsbS and NPQ capacity may have a more important role in species such as rice that are adapted to higher light intensities (Hubbart et al. 2012).

Photoinhibitory losses occur at high radiation when yield potential is also highest. However this yield potential is still below theoretical maximum, one of the mechanisms being photoinhibition (Zhu et al. 2008b; Zhu et al. 2010). Depression in the photosynthetic capacity in rice can be observed in the field when irradiance exceeds saturation levels (around 1000 μmol m^−2^ s^−1^) (Black et al. 1995; Murchie et al. 1999; Wang et al. 2005). Indeed it is important to realise that such chronic photoinhibition in tropical rice exists even under optimal conditions in the field, as displayed by a significant decline in the quantum yield of carbon fixation at midday. In this scenario, the plants may initially experience high levels of qE only to shift to a more chronic form (qI) under increasing and sustained exposure to higher solar irradiances and/or other abiotic stresses. This midday depression results in an estimated loss of 30 % of photosynthesis during the reproductive stage (Murchie et al. 1999; Horton 2000). This is often but not always associated with other metabolic factors including decreased stomatal conductance, an accumulation of carbohydrates and a decrease in ΦPSII. Despite being a rainfed and irrigated crop, rice can be significantly affected by other environmental disturbances including periodic drought and submergence in lowland plantations in South and Southeast Asia (Wade et al. 1999; Zhou et al. 2007), caused by climate-related events such as El Niños. Heat stress is also common under natural field conditions, where temperatures and light intensities can reach 40 **°**C and beyond 1500 μmol m^−2^ s^−1^ respectively (Jagadish et al. 2010). Nutrient deficiency can also have an effect on photosynthetic capacity and susceptibility to photosynthesis in rice (Chen et al. 2003; Herman et al. 2015). All of these factors will compound the impact of photoinhibition.

#### The way Forward: Improving Canopy Photoprotection to Improve Yield

Exploitation of rice genetic resources may be limited by technical problems with the routine high throughput measurement of physiological traits such as NPQ and photoinhibition. Although both are easily measured by hand there is a required period of dark adaptation which can make application to mutant populations (tens of thousands of plant lines) difficult. However, the continued development of high-resolution phenotyping platforms (with automated darkening and light treatment) is expected to overcome this and uncover the genetic basis of these traits used in breeding programmes (Furbank and Tester 2011; Murchie and Harbinson 2015).

Photoprotection at the canopy level has not been fully addressed and there are outstanding questions concerning optimisation of photoprotection for canopy photosynthesis in the field (Murchie et al. 2009; Zhu et al. 2010). Intuitively, protection has beneficial effects to growth and yield because it reduces the likelihood of photoinhibition and photooxidative stress. Such effects have been shown for model species such as *A. thaliana* with varying levels of PsbS (Frenkel et al. 2009) but evidence for crops in the field is limited. Additionally the potential has been shown for over ‘expression’ of qE to result in down regulation of photosynthesis in fluctuating light which suggests that the reduction on quantum yield inherent within photoprotection creates a trade-off between control of oxidative stress and productivity (Figure 9 in Hubbart et al. 2012). To disentangle these effects to understand how photoprotection is involved in yield formation in rice we must consider photoprotective mechanisms solely in response to irradiance. We note there has been interesting recent work suggesting that NPQ is involved in the regulatory cross – talk in signalling responses to biotic factors. For example NPQ suppresses ROS formation and evidence shows that this can lead to plants being more palatable to herbivores (Jankanpaa et al. 2013). Recent work has suggested that activation of early defense reponses by pathogen –associated molecular patterns (PAMPS) triggers a temporary reduction in NPQ in order to permit and possibly as part of ROS mediated signalling and plant immunity (Göhre et al. 2012).

The lack of an empirical link between photoprotection and yield requires more explanation. First, it can be difficult to isolate photoprotective mechanisms as the origin of variations in productivity and yield. Photosynthesis has many mechanisms that can compensate for genetically induced variation in photochemical and non-photochemical processes. Indeed NPQ can acclimate to the light environment and has a regulatory role in buffering the electron transport chain against rapid fluctuations in light (e.g. Murchie and Harbinson 2015). The use of knockout lines and overexpressors has demonstrated the role of some photoprotective processes in rice but impact at field and whole plant level has rarely been shown although we note studies that use additional abiotic stress such as drought (e.g. Du et al. 2010).

Conceptually there is a problem in quantifying ‘how protective’ each process is (Ruban and Murchie 2012). Usually one would use control plants with no altered protection and compare to mutants or transgenics with a well-characterised genetic alteration. However it will be necessary to deconvolute the different mechanisms of photoprotection in the field to identify which compensatory processes are in action and this is technically difficult at the canopy scale. There are suggestions that photoinhibition in natural plant populations may occur as a result of down-regulation of photosynthesis which has itself been caused by carbohydrate accumulation (Adams et al. 2013), obscuring the effect of photoinhibition on photosynthesis. However no such effects have been observed in crops: in fact a large accumulation of leaf carbohydrate did not correlate with photoinhibition and photosynthesis (Murchie et al. 2002) so this mechanism does not appear to be present, in rice at least. Ultimately the role of photoprotection in crop yield will need to be demonstrated using canopy level practices such as those for measuring radiation use efficiency (Sinclair and Muchow 1999; Murchie and Reynolds 2012).

Second, crop canopy structure may have a substantial role. It defines the characteristics of light in space and time and can offer properties that both enhance and help to prevent photoinhibition. In most rice crop canopies the uppermost layers receive the highest radiation. Thus in dense canopies, upper layers may offer protection to those leaves lower down. Unfortunately, this is at the risk of productivity because upright leaves with efficient penetration are associated with the highest canopy photosynthesis rates (Sinclair and Muchow 1999; Murchie and Reynolds 2012). This is because erect leaved canopies also permit an accumulation of a high leaf area index and a more efficient distribution of photosynthesis by prevention of a high proportion of leaves forming a light-saturated state on the one hand or being overly light-limited on the other (Burgess et al. 2015). Such erect leaved canopies possess higher theoretical canopy photosynthetic rate and reduced risk of photoinhibition at midday (Murchie et al. 1999; Sinclair and Muchow 1999). This point is important because other yield components are close to optimisation (such as harvest index) and canopy photosynthesis is currently considered a significant trait in terms of enhancing rice yield (Murchie et al. 2009, Zhu et al. 2010).

Modelling of dynamic photoinhibition has shown that the complex 3D structure of crop canopies can result in significant reductions in carbon gain by virtue of the failure of photosynthesis to efficiently track the fluctuations in light that occur over a timescale of minutes and hours (Zhu et al. 2004; Song et al. 2013). This has also been shown using stomatal dynamics (Lawson and Blatt 2014). To emphasise, a denser canopy with a high extinction coefficient would reduce the occurrence of such fluctuations but at the risk of reducing potential productivity.

There are therefore likely to be a series of optimisations, unique for photoprotection, that operate according to canopy structure. It is possible to hypothesise that more photoprotection will be required in some circumstances (prolonged high light) and less under others (low light or during periods of moderate but rapid high – low transition). The patterns of photoprotection required will be determined by the proportion of canopy surface area exposed to high radiation that induces photoinhibition and the properties of light fluctuations that limit photosynthesis. So how can this problem be solved? Existing models of canopy photoinhibition can predict the impact of ΦCO_2_ changes on canopy photosynthesis, using different approaches such as ray tracing within high resolution images of crop canopy structure to track light fluctuations in time according to canopy position (e.g. Ogren 1993; Werner et al. 2001; Zhu et al. 2004; Burgess et al. 2015). High throughput, high-resolution techniques for canopy imaging are under development and success has been achieved (Pound et al. 2014). These empirical approaches are essential for establishing the impact on yield but in order to predict and test the impact of individual components and to integrate these processes into wider photosynthetic metabolism it will be necessary to generate mechanistic models of both photoinhibition and photoprotection based on component kinetics. However we are a long way from models that are sufficiently parameterised to make this link despite recent progress at the thylakoid level (Zaks et al. 2012). The focus needs to be on linking dynamic canopy descriptions with metabolic models that integrate photoprotective processes with complete models of photosynthesis (Song et al. 2013; Zhu et al. 2013; Burgess et al. 2015). Predictions can then be tested using transgenic, mutant or introgression/wide crossing approaches.

### Conclusion: Towards an Uncertain Future

The genetic improvement of crop yield will need to be integrative and consider multiple stress and signalling pathways in the plant. In this review we focus on responses to high light, which will compound the effects of other stresses. We have highlighted the weak links between leaf level effects and field yield and explain the case for a better understanding of canopy level optimisation.

Finally we note some novel and highly relevant climatic factors related to irradiance. Studies carried out since the 1990s found a decline in solar radiation at the surface of the earth between the 1950s and the 1980s (Wild and Wild 2009), a phenomenon now commonly identified as ‘global dimming’. Recent studies have noted a trend reversal post 1980, known as ‘global brightening’. Changes in atmospheric aerosols caused by pollution have been recognized as one of the key contributors in these effects (Ramanathan and Carmichael 2008). We propose that changes in future global radiation need to be considered carefully because they may represent a further need for photoprotection in areas where radiation is highest.

## Abbreviations

ATP: Adenosine Triphosphate
CO_2_: Carbon Dioxide
GR: Glutamate Reductase
H^+^: Hydrogen Ions
H_2_O_2_: Hydrogen Peroxide
NADP^+^: Nicotinamide Adenine Dinucleotide Phosphate
NADPH: Reduced Nicotinamide Adenine Dinucleotide Phosphate
NPQ: Non-photochemical Quenching
P_max_: Light-saturated Rate of Photosynthesis
PSI: Photosystem I
PSII: Photosystem II
ROS: Reactive Oxygen Species
qE: Rapid and Reversible form of Non-photochemical Quenching
qI: Sustained, Long Term form of Non-photochemical Quenching
SOD: Superoxide Dismutase
UV: Ultraviolet Radiation
XC: Xanthophyll Cycle
ΔpH: Change in pH Across the Thylakoid Lumen
ΦCO_2_: Quantum Yield of Carbon Dioxide Assimilation
ΦPSII: Operational Efficiency of Photosystem II
•O_2_^−^: Superoxide Radical
